# Regulatory Interactions between Androgens, Hoxb5, and TGF****β**** Signaling in Murine Lung Development

**DOI:** 10.1155/2013/320249

**Published:** 2013-09-03

**Authors:** MaryAnn V. Volpe, Sujatha M. Ramadurai, Sana Mujahid, Thanhxuan Vong, Marcia Brandao, Karen T. Wang, Lucia D. Pham, Heber C. Nielsen

**Affiliations:** ^1^Division of Newborn Medicine, Department of Pediatrics, Floating Hospital for Children at Tufts Medical Center and Sackler School of Biomedical Sciences, Tufts University School of Medicine, Boston, MA 02111, USA; ^2^Division of Neonatology, Department of Pediatrics, Tufts Medical Center, Boston, MA 02111, USA

## Abstract

Androgens enhance airway branching but delay alveolar maturation contributing to increased respiratory morbidity in prematurely born male infants. Hoxb5 protein positively regulates airway branching in developing lung. In other organs, androgen regulation intersects with Hox proteins and TGF**β**-SMAD signaling, but these interactions have not been studied in the lung. We hypothesized that androgen alteration of airway branching early in lung development requires Hoxb5 expression and that these androgen-Hoxb5 interactions occur partially through regional changes in TGF**β** signaling. To evaluate acute effects of androgen and TGF**β** on Hoxb5, E11 whole fetal mouse lungs were cultured with dihydrotestosterone (DHT) with/without Hoxb5 siRNA or TGF**β** inhibitory antibody. Chronic *in utero* DHT exposure was accomplished by exposing pregnant mice to DHT (subcutaneous pellet) from E11 to E18. DHT's ability to enhance airway branching and alter phosphorylated SMAD2 cellular localization was partially dependent on Hoxb5. Hoxb5 inhibition also changed the cellular distribution of SMAD7 protein. Chronic *in utero* DHT increased Hoxb5 and altered SMAD7 mesenchymal localization. TGF**β** inhibition enhanced airway branching, and Hoxb5 protein cellular localization was more diffuse. We conclude that DHT controls lung airway development partially through modulation of Hoxb5 protein expression and that this level of regulation involves interactions with TGF**β** signaling.

## 1. Introduction

Hox transcription factors regulate expression of specific genes essential to normal embryogenesis, organogenesis, and maintenance of cell fate and differentiation [1–4]. We and others have established the importance of the Hox protein Hoxb5 in fetal lung airway branching [5–13]. These studies show that Hoxb5 has unique temporal, spatial, and cellular expression patterns that change with progressive stages of lung development. In both murine and human lung, Hoxb5 is expressed in lung fibroblasts beginning at the earliest stages of lung branching morphogenesis, peaking at the end of the pseudoglandular stage. Thereafter, Hoxb5 expression diminishes as branching morphogenesis is completed and saccularization with subsequent alveologenesis commences. The downregulation of Hoxb5 in late gestation mouse lung development also exhibits a sex difference, occurring later in males than in females [8]. Growth factors and steroid and thyroid hormones that enhance alveolar maturation downregulate Hoxb5 [14–16]. Hoxb5 regulates airway branching *in vitro* in murine embryonic lung bud cultures. It is upregulated in the human congenital anomalies bronchopulmonary sequestration (BPS) and congenital cystic adenomatoid malformation (CCAM), diseases associated with aberrantly increased airway branching [9–11]. 

In the developing lung, androgens stimulate airway branching morphogenesis and cell proliferation but delay alveolar epithelial maturation, thereby contributing to the increased respiratory morbidity in prematurely born male infants [17–21]. The ability of androgens to influence airway branching and lung maturation occurs through effects on lung fibroblasts and subsequent changes in fibroblast-epithelial cell communication [19, 22, 23]. How androgens affect Hoxb5, a transcription factor that is exclusively expressed in the mesenchyme and necessary for airway branching, is not known. This is important to know because androgen effects in the prostate, another organ that undergoes an endodermal branching process, are mediated by Hox transcription factor proteins where the Hox protein actions are necessary for normal androgen signaling. Further, Hox gene expression is altered in prostate cancer [24]. These Hox-androgen interactions are complex. While androgens can regulate Hox proteins, Hox proteins can suppress androgen receptor expression by directly binding to the androgen receptor promoter [24, 25]. 

Some of the effects of androgens on lung development likely involve gestational age-dependent changes in TGF*β*  signaling as well [23, 26–28]. In the prostate, androgens mediate TGF*β*  signaling to coordinate organ morphogenesis and tissue homeostasis. Uncoupling of androgen-TGF*β* signaling leads to prostate abnormalities including cancer [28]. At a molecular level, activated androgen receptors bind as cofactors with TGF*β* receptor-activated SMADs. SMADs also act as repressors or activators of androgen receptor activity [26, 29, 30]. TGF*β* signaling is also modulated by Hox proteins that act as SMAD cofactors to help initiate or repress downstream signaling events [31–36]. 

Despite the evidence for these regulatory interactions between androgens, Hox proteins and TGF*β* signaling in other branching organs and knowledge of the individual importance of androgen, Hox proteins, and TGF*β* in lung development and disease, the role of interactions between androgen, Hox proteins, and TGF*β* signaling in developing lung has not been studied.

The overall objective of these studies was to begin to develop an understanding of the role of androgen-Hoxb5 mediated effects in the developing lung and whether these mechanisms involve changes in TGF*β* signaling. We hypothesized (1) that the ability of androgen exposure to alter airway branching early in lung development requires Hoxb5 expression and regulation and (2) that these androgen-Hoxb5 interactions occur partially through regional changes in TGF*β* signaling. 

## 2. Materials and Methods

### 2.1. Animals

The animal study protocol was approved by the Institutional Animal Research Committee (IACUC). Principals of laboratory animal care as outlined by the National Institutes of Health Guidelines for Care and Use of Laboratory Animals (National Institutes of Health publication 86-23, revised 1985) were followed. Timed pregnant Swiss Webster mice were obtained from Charles River Laboratories (Wilmington, MA, USA) with the morning of the vaginal plug defined as Gestational Day 0 (E0, term is 19 days).

### 2.2. *Ex Vivo* Whole Fetal Lung Cultures

 E11 fetal mouse lung cultures were prepared as we have previously described [10, 11, 18]. E11 whole fetal lungs were placed on GVWP membranes (Millipore, Bedford, MA, USA) suspended at the air-liquid interface on metal grids in 35 mm culture dishes and cultured in a 37°C, 21% O_2_, 5% CO_2_ incubator with DMEM containing 10% charcoal stripped FCS (FCS-) with penicillin (50 units)/streptomycin (50 *μ*g)/mL and used for the following experiments.

### 2.3. DHT Exposure and RNAi Inhibition of Hoxb5

 The Hoxb5 siRNA sequence, siRNA control sequences (Ambion), and concentrations and transfection conditions used to block Hoxb5 translation were identical to those used in our previous studies where we performed a dose-range study determining that 200 nM Hoxb5 siRNA significantly decreased Hoxb5 protein concentration by 50% compared to scramble control siRNA [10, 37]. SiRNA molecules were complexed with Transit-TKO (Mirus, Madison, WI, USA) as per manufacturer's recommendations. Starting at the initiation of the culture period, lung organ cultures were randomly assigned to the following treatment conditions: control (no treatment); dihydrotestosterone (DHT 10^−7^ M); and the following conditions with or without added DHT (10^−7^ M): siRNA targeting Hoxb5 (200 nM); scrambled siRNA (200 nM, negative control); or transfection vehicle alone (*N* = 3–8 independent experiments) [18, 27, 38]. Media and reagents were not changed during the culture period. Previous experiments from our lab documented that the ethanol diluent (<1% concentration) for the DHT or gender did not affect the response of E11 cultured mouse lungs to DHT [18]. Therefore, we did not include separate cultures with ethanol alone or identify gender in these experiments. 

### 2.4. Inhibition of TGF*β*

In preliminary experiments we performed dose response studies of the 1d11 pan-specific TGF*β* antibody (Genzyme, Boston, MA, USA) or MOPC21 isotype matched IgG antibody (Sigma, St. Louis, MI, USA) at 1–100 ug/mL concentration (data not shown) [39]. A TGF*β* antibody dose of 10 ug/mL treatment at start of culture and after 48 hr produced effects on lung development and TGF*β* signaling without toxicity and was therefore used in all subsequent experiments. Cultures were randomized to (1) TGF*β* inhibitory antibody (10 ug/mL of 1D11); (2) MOPC21 antibody control (10 ug/mL); or (3) no treatment (*N* = 6–17 lungs per condition from 4 independent experiments). 

### 2.5. Quantification of Branching Morphogenesis and Velocity of Branching by Terminal Bud Counts

 Each lung was evaluated for changes in lung morphology and airway branching at 24 hour intervals in culture using a Nikon inverted diaphot light microscope. Branching morphogenesis was measured as we have previously described [11, 38]. Briefly, for each lung in which the right and left lungs were intact and the left lung could be identified by its branching pattern, the numbers of terminal buds (most distal branch of each airway generation) in the left lung at 24, 48, and 72 hours were counted. The velocity of airway branching of each lung explant was determined by recording the change in airway branch numbers in each lung explant compared to itself from the previous day of culture (*N* = 9–22 lungs per condition from 6 separate experiments) [11, 18].

### 2.6. *In Vivo* DHT Exposure

Chronic *in utero* DHT exposure was accomplished as we have previously described by implanting time-dated pregnant Swiss Webster mice on E11 (term is 19 days) with subcutaneous time-released DHT pellets (2 mg/day). This DHT dose was used with a goal of inducing androgen-induced lung effects on both sexes as we have previously described [17, 19, 27]. Control (sham surgery) animals were operated on in the same manner as animals where the DHT pellet was surgically implanted. At E18, animals were sacrificed by CO_2_ inhalation with cervical dislocation. The fetal lungs were removed under sterile conditions and processed for immunohistochemistry and western blot analyses as described next (*N* = 4 experiments with fetal lungs from five pregnant mice/experiment).

### 2.7. Immunohistochemistry

Sequential coronal lung cryosections (6 microns) from control and DHT treated lungs were prepared as we have previously described [9–11] and immunostained for Hoxb5 [11], phosphorylated SMAD2 (SMAD2P), and total SMAD7 (cell signaling). Sections were incubated overnight (4°C) with either Hoxb5 (1/300), SMAD2P (1/200), or SMAD7 (1/800) primary antibodies, followed by room temperature incubation with corresponding secondary antibody (1/200) and avidin-biotin complex conjugated to alkaline phosphatase. Blue alkaline phosphatase chromogen plus levamisole (endogenous alkaline phosphatase blocker) were used for alkaline phosphatase detection, followed by Fast Red counterstaining. Secondary antibodies, immunostaining kits and reagents were from Vector (Burlingame, CA, USA). Lung sections from all experimental conditions were incubated for the same period of time in the alkaline phosphatase detection solution. Each immunostaining experiment (*N* ≥ 3 for each antibody) included controls of adjacent sections with omission of respective primary antibody. No staining was seen in the absence of primary antibody, as we have previously shown [8, 11, 16].

### 2.8. Western Blot and Densitometry

Control and DHT treated lungs from *in vivo* studies (*N* = 4 separate experiments, 3–6 lungs per experiment) were prepared for western blots as we have previously described [10, 11]. Briefly, proteins were first homogenized in the presence of proteinase and phosphatase inhibitors with protein concentration determined by the Lowry method. Twenty micrograms of total lung protein for each sample were loaded onto polyacrylamide gels and proteins separated by SDS-PAGE. After SDS-PAGE, proteins were transferred to nitrocellulose membranes by wet transfer followed by incubation with Hoxb5 antibody, HRP-linked secondary antibody (Jackson ImmunoResearch, West Grove, PA, USA), and subsequent chemiluminescence detection (Perkin Elmer, Boston, MA, USA) [10]. Each membrane was then stripped and reprobed with GAPDH or *β*-actin primary antibody (Ambion, Austin, TX). Hoxb5 densitometric results (Alpha Innotech, San Leandro, CA, USA) were normalized to GAPDH densitometry values as internal control for each blot [10].

### 2.9. Statistical Analysis

Terminal bud counts and the velocity of airway branching between experimental groups were statistically compared using nonparametric ANOVA with Kruskal Wallace post hoc testing for specific differences (Instat, Graphpad Software, San Diego, CA, USA). Densitometry results were evaluated by Student's *t*-test with Welch's corrections. For immunostaining, lung sections from at least three lungs per experimental condition were visually assessed and compared by light microscopy. 

## 3. Results

### 3.1. Androgen Alters Airway Branching by Modulating Hoxb5 Mesenchymal Expression and TGF*β* Signaling

**Figure 1:**
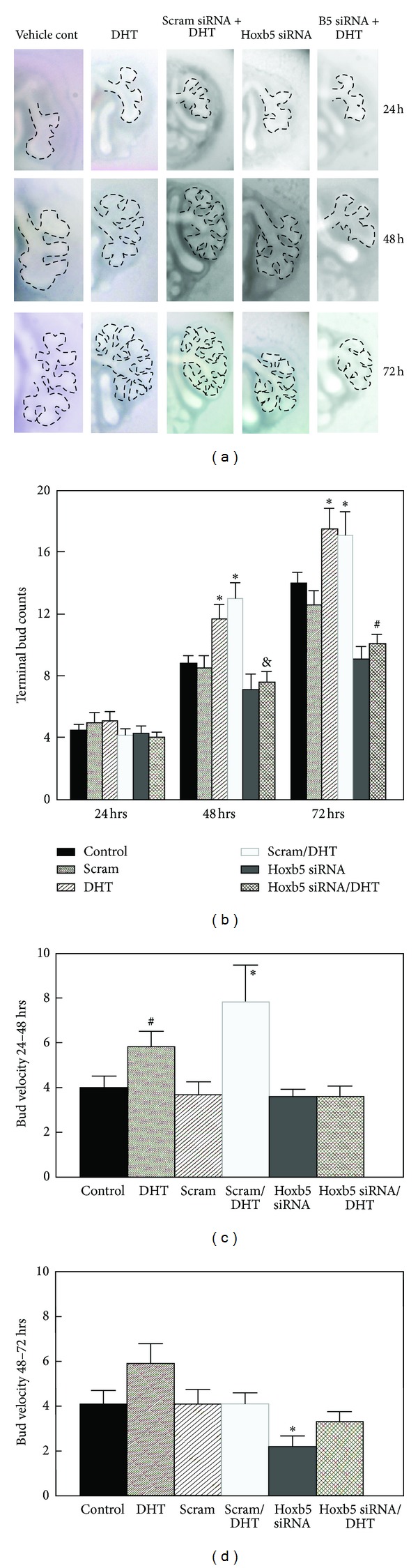
Effect of DHT without and with Hoxb5 knockdown on airway branch development of cultured E11 whole fetal lungs. Images are high-power views (10x Mag) of the left lung of representative *ex vivo* whole lungs in culture photographed at 24 hour intervals. The dashed lines outline airway branches. Whole lung images are shown in Supplementary Figure 1 (Supplementary Matrial available online at http://dx.doi.org/10.1155/2013/320249). (a) Over the 72 hours of culture all lungs continued to branch, but DHT treatment (DHT and scram/DHT) increased airway arborization compared to control lungs. Hoxb5 siRNA transfection resulted in a disorganized and reduced branching pattern compared to control and DHT-treated lungs. This effect of Hoxb5 siRNA remained even in presence of DHT. (b) Terminal bud counts confirmed that all lungs continued to branch over time in culture. Terminal bud counts at 24 hours of culture were similar in all experimental conditions. At 48 and 72 hours, terminal bud counts were significantly greater in DHT and scramble + DHT treated lungs compared to control and scramble treated lungs (*P ≤ 0.04, *N* = 9–22, mean ± SEM, control versus DHT and scram versus scram/DHT). Hoxb5 knockdown reduced airway branching at 48 hr compared to scramble control. This difference became significant at 72 hr (^#^ *P* = 0.01, *N* = 9–22, mean ± SEM, scram versus Hoxb5 siRNA at 72 hr). Hoxb5 knockdown prevented airway branching induced by DHT treatment at 48 and 72 hours culture (scram/DHT versus Hoxb5 siRNA/DHT, ^&^ *P* = 0.04 at 48 hr; ^#^ *P* = 0.02 at 72 hr, mean ± SEM, *N* = 9–22 lungs). (c) The velocity of branching (bud velocity) from 24–48 hours was significantly increased with DHT treatment (mean ± SEM; ^#^ *P* = 0.01, control versus DHT; *P = 0.02, scram/DHT versus Hoxb5 siRNA/DHT). (d) The velocity of branching from 48 to 72 hours was significantly inhibited by Hoxb5 knockdown (mean ± SEM; *P = 0.04, Hoxb5 siRNA versus scram).

To determine the potential for Hoxb5 protein to regulate androgen effects on airway branching, we cultured E11 mouse fetal lungs with DHT (10^−7 ^M) in the presence or absence of Hoxb5-specific siRNA targeting Hoxb5 protein expression.  Figure 1 demonstrates high-power view of the left lung of representative explants with dashed lines used to outline airway branches. Whole lung images are included in Supplementary Figure 1. All lungs had a similar pattern of branching at the start of culture (3 left lung terminal airway branches; data not shown) and after 24 hours (Figure 1(a)). However, over time in culture DHT-treated lungs (DHT and scram siRNA/DHT) developed a more complex branching pattern with apparently more numerous and densely packed branches compared to control lungs without DHT treatment. The increased branches seen with DHT exposure appeared to have normal morphology. Hoxb5 siRNA treated lungs developed short, widened, and less-organized branching similar to what we have previously reported with Hoxb5 siRNA inhibition [10, 11]. DHT treatment in the presence of Hoxb5 siRNA did not overcome the inhibitory effect of Hoxb5-siRNA on airway branching indicating that RNAi knockdown of Hoxb5 prevented DHT effects on airway branching. 

These visual changes in airway branching were quantified by terminal bud counts and velocity of airway branching (Figures 1(b), 1(c), and 1(d)). Compared to no treatment (control) and scrambled siRNA treated lungs, terminal bud counts at 48 and 72 hr of culture (Figure 1(b)) were significantly increased in lungs treated with DHT without or with the addition of scrambled control siRNA. However, Hoxb5 siRNA treated lungs had minimally increased airway branch numbers, and terminal bud counts were significantly less than vehicle and scrambled treated lungs. Further, the ability of DHT to increase airway branching was prevented by Hoxb5 siRNA treatment. To determine the individual effects of each treatment on each individual lung, we calculated the velocity of branching as the change in airway branching in each lung compared to itself over time in culture. The velocity of branching (Figure 1(c)) from 24 to 48 hr was significantly increased by DHT, both without and with scrambled siRNA treatment (DHT with 6  ±  0.5 terminal lung branches versus scramble + DHT with 7.7  ±  1.6 terminal branches). The progressive inhibition of airway branching in Hoxb5 siRNA treated lungs led to significantly decreased velocity of budding at 72 hr (Figure 1(d)). This effect of Hoxb5 inhibition was blunted but not eliminated by DHT treatment (Figure 1(d)). There was no change in the velocity of branching in scrambled and scrambled/DHT conditions at 72 hours. It is likely that the majority of the increased arborization of the airway tree induced by DHT occurred in the first 48 hours after addition of DHT. This is a similar response to DHT treatment as in our first study evaluating DHT effects on *in vitro* airway branch development [18]. 

**Figure 2:**
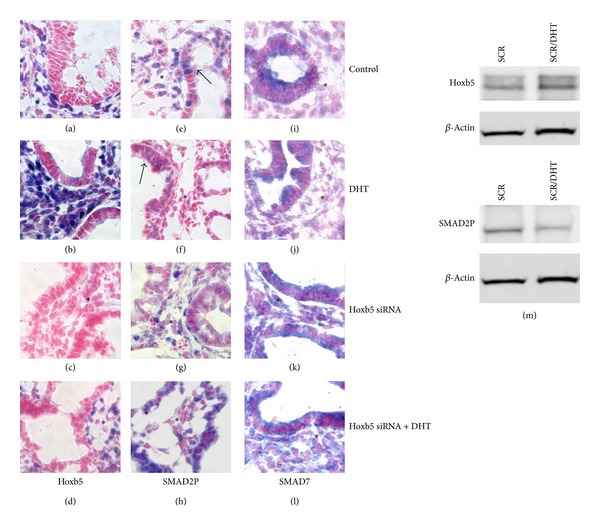
Effect of DHT without and with Hoxb5 knockdown on lung histology and Hoxb5, SMAD2P, and SMAD7 spatial and cellular protein expression. (a–l) Immunohistochemistry: (a–d) Hoxb5; (e–h) SMAD2P; (i–l) SMAD7; (m) Hoxb5 and SMAD2P western blot analyses in DHT-treated lungs. Asterisk (∗) represents mesenchyme regions of lung in all tissue sections. Blue staining represents specific staining for protein of interest; red staining is Texas Red counterstain. Compared to control treated lung (a), DHT led to more diffusely intense Hoxb5 mesenchymal (∗) localization (b) and increased Hoxb5 protein levels (m). Hoxb5 knockdown profoundly diminished Hoxb5 staining (c) and partially prevented the ability of DHT to alter Hoxb5 mesenchymal (∗) cell protein expression (d). DHT treatment diminished SMAD2P mesenchymal (∗) and epithelial staining (arrows in (f) compared to control (e)) and total protein levels (m). With knockdown of Hoxb5 expression (g, h) SMAD2P staining was more diffuse than with DHT treatment alone ((h) compared to (f)). In the presence of Hoxb5 knockdown, DHT lost the inhibitory effect on SMAD2P protein (h) cellular expression. SMAD7 expression was not changed by DHT treatment (j), whereas Hoxb5 knockdown changed the mesenchymal (∗) distribution of SMAD7 (k). This effect of Hoxb5 knockdown on SMAD7 persisted in the presence of DHT (l). 100x Mag; representative tissue sections from at least 3 lungs for each condition from 3 experiments. Immunostaining for different experimental conditions was done in simultaneous reactions and incubated for the same time periods, allowing direct comparison of spatial and cellular localization of the proteins studied.

Compared to control lungs (Figure 2(a)), lungs treated with androgen had more diffuse Hoxb5 protein mesenchymal staining around branching airways (Figure 2(b)), and DHT increased protein levels of Hoxb5 within these lungs (Figure 2(m)). However, Hoxb5 siRNA treatment led to a paucity of Hoxb5-positive cells in a less dense mesenchyme surrounding dilated airways that reached the peripheral margins of the lung (Figure 2(c) and Supplementary Figure 2). These findings concerning Hoxb5 siRNA inhibition and altered airway branching are consistent with our previous studies in *ex vivo* whole fetal mouse lungs, where we demonstrated that Hoxb5 inhibition with the same Hoxb5 siRNA sequence decreased Hoxb5 protein levels by 50% compared to scramble control treatment [10, 11]. DHT treatment in the presence of Hoxb5 siRNA did not completely overcome the siRNA-induced inhibition of Hoxb5 protein expression (Figure 2(d) compared to Figure 2(b)), and airway morphology retained the characteristics seen with Hoxb5 siRNA treatment (see Supplementary Figure 2).

We evaluated the effect of androgen combined with Hoxb5 knockdown on the expression pattern of SMAD2P and SMAD7 proteins in these lungs. SMAD2P is an important effector of TGF*β* signaling in lung development and is negatively modulated by SMAD7 [40, 41]. Compared to control (Figure 2(e)), DHT treatment led to less-evident mesenchymal and epithelial SMAD2P protein nuclear localization (Figure 2(f)) and reduced SMAD2P total protein levels (Figure 2(m)). Hoxb5 knockdown prevented the DHT-induced changes in mesenchymal and epithelial SMAD2P localization (Figure 2(h) compared to Figure 2(f)). DHT did not appreciably change SMAD7 staining or cellular localization (Figure 2(j) versus control, Figure 2(i)). Hoxb5 knockdown, however, appeared to alter the localization of SMAD7 in lung mesenchyme (Figure 2(k)). This change in SMAD7 mesenchymal localization continued when Hoxb5 knockdown lungs were also treated with DHT (Figure 2(l)). These results indicate that Hoxb5 contributes to androgen control of airway branching and suggest that DHT effects on TGF*β* signaling may be partially controlled by androgen induction of Hoxb5 and subsequent changes in SMAD2P and SMAD7 cellular localization. We did not observe any changes in SMAD2 spatial and cellular localization (data not shown).

### 3.2. Effects of TGF*β* Inhibition on Airway Branching and Cellular Localization of SMAD2P, SMAD7, and Hoxb5 Proteins

**Figure 3:**
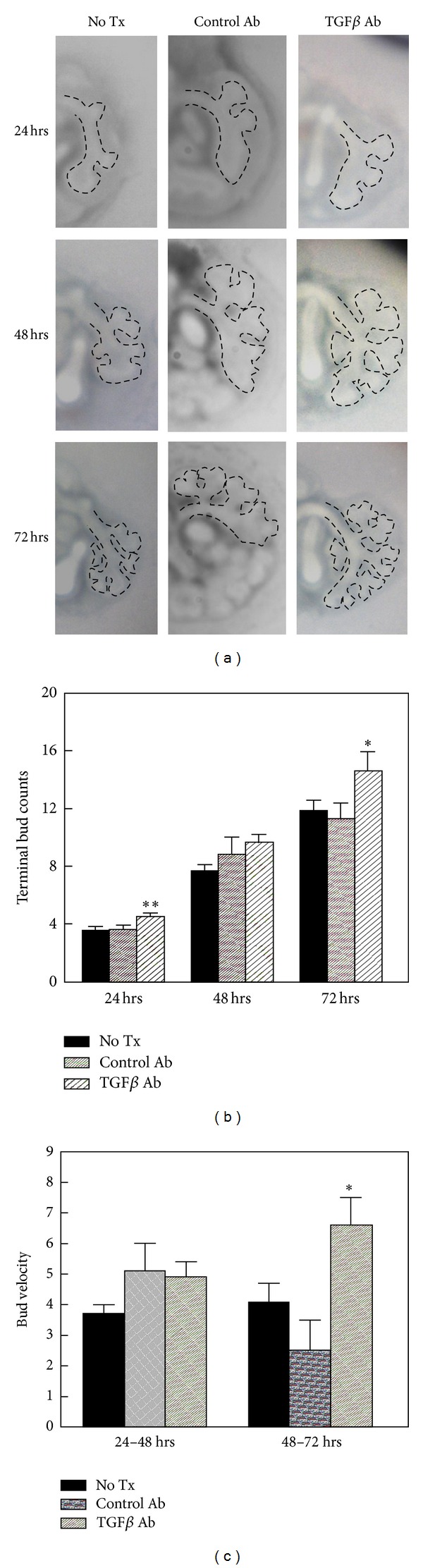
Effect of TGF*β* inhibition on airway branch development in cultured E11 whole fetal lungs. (a) Images are high-power views of the left lung of representative *ex vivo* whole lungs in culture photographed at 24-hour intervals. The dashed lines outline airway branches. Over time in culture, lungs in all three conditions had progressive branching. However, at 48 and 72 hours the lungs treated with TGF*β* inhibitory antibody had more developed airway arborization, and these branches appeared normal in size and shape compared to no antibody and control antibody treated lungs. (b) Terminal bud counts confirmed that airway branch numbers were significantly increased by TGF*β* inhibitory antibody treatment after 24 and 72 hours of culture (**P = 0.02, *P = 0.03, TGF*β* Ab versus control antibody and no Tx, mean ± SEM, *N* = 6–17 lungs). (c) The velocity of branching was significantly increased with TGF*β* antibody treatment (*P = 0.01, TGF*β* Ab versus control Ab and no Tx, mean ± SEM).

Developmental signaling pathways for androgens, Hox proteins, and TGF*β* each have feedback loops to modulate their downstream signaling [24, 25, 31, 32, 42]. For this reason, we studied whether TGF*β* inhibition alone affects airway branching and whether this change in airway branching involves TGF*β* regulation of Hoxb5. As effector SMAD phosphorylation is the major pathway mediating TGF*β* effects, we evaluated SMAD2P as an indicator of TGF*β* signaling activity. E11 mouse fetal lungs cultured with the TGF*β* inhibitory antibody (1d11, Genzyme) had apparent increased airway arborization over 72 hours in culture (Figure 3(a), left lung high-power view with outlined airway branches) and appeared larger (Supplementary Figure 3(a)). Compared to both no treatment and control antibody treated cultures, terminal bud counts (Figure 3(b)) were significantly increased in TGF*β*-antibody treated cultures at 24 and 72 hours culture. This indicates that the effect of the TGF*β* antibody was most evident 24 hours posttreatment (24 hours after initial treatment at onset of culture and after treatment at 48 hours of culture). The velocity of branching (Figure 3(c)) was significantly increased after 72 hr of culture in TGF*β* antibody treated lungs. 

**Figure 4:**
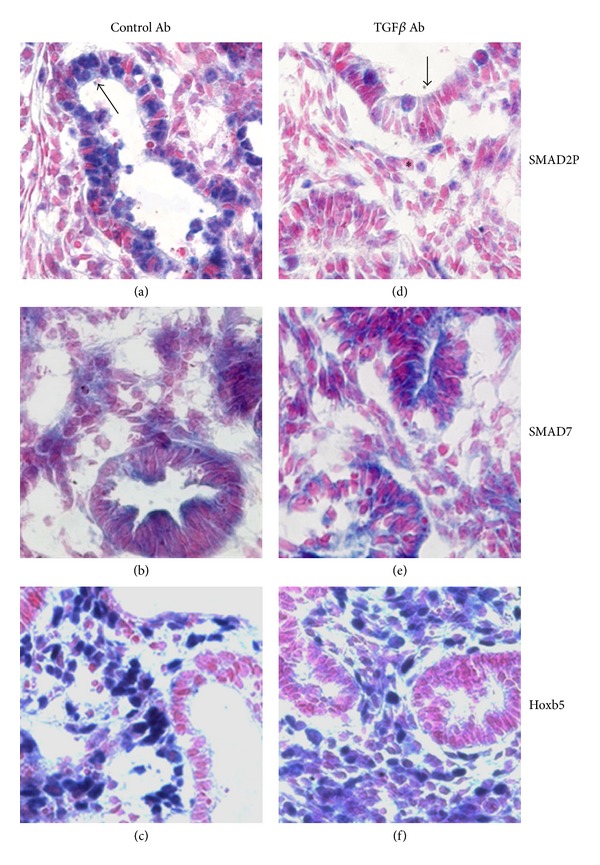
Effect of TGF*β* inhibition on lung histology and Hoxb5, SMAD2P, and SMAD7 spatial and cellular expression. Cultured E11 fetal mouse lungs treated with control antibody had similar staining for SMAD2P (a), SMAD7 (b), and Hoxb5 (c) as untreated controls (Cf. Figure 2). Inhibitory TGF*β* antibody (1d11) treatment led to formation of immature appearing airways that mostly had narrow lumens and decreased epithelial (↑) and mesenchymal (∗) SMAD2P immunolocalization (d), whereas SMAD7 (e) was relatively unchanged. Hoxb5 (f) mesenchymal localization appeared more diffuse with TGF*β* antibody treatment. Figures are representative tissue sections from at least 3 lungs per condition from 3 experiments; 100x Mag.

Tissue histology revealed that TGF*β* inhibition led to more airways having tall columnar epithelium and narrow airway lumens, morphology typical of less mature airways (Figure 4) [43]. We did not see any changes in epithelial distribution of Sox2 protein suggesting that there was no significant effect on proximal-to-distal epithelial differentiation at this stage of development (data not shown). Inhibition of TGF*β* signaling led to less-evident mesenchymal and epithelial staining for SMAD2P (Figure 4(d)) but did not appreciably change SMAD7 (Figure 4(e)) cellular staining or localization. Hoxb5 mesenchymal cell localization appeared more diffuse in lungs treated with TGF*β* inhibitory antibody (Figure 4(f)).

### 3.3. Androgen Affects Hoxb5 and SMAD Regulation during *In Vivo* Lung Development

Previous studies from our lab have shown that *in vivo* exposure to androgens beginning with the start of sexual differentiation leads to delayed and altered lung development and maturation in female fetuses, resembling a maturational timetable more similar to males [19]. Using our established *in vivo* androgen exposure model, we also examined how androgen exposure during *in utero* lung development alters Hoxb5 protein levels and cellular expression and whether these changes correlate with changes in effector and inhibitory SMADs. 

**Figure 5:**
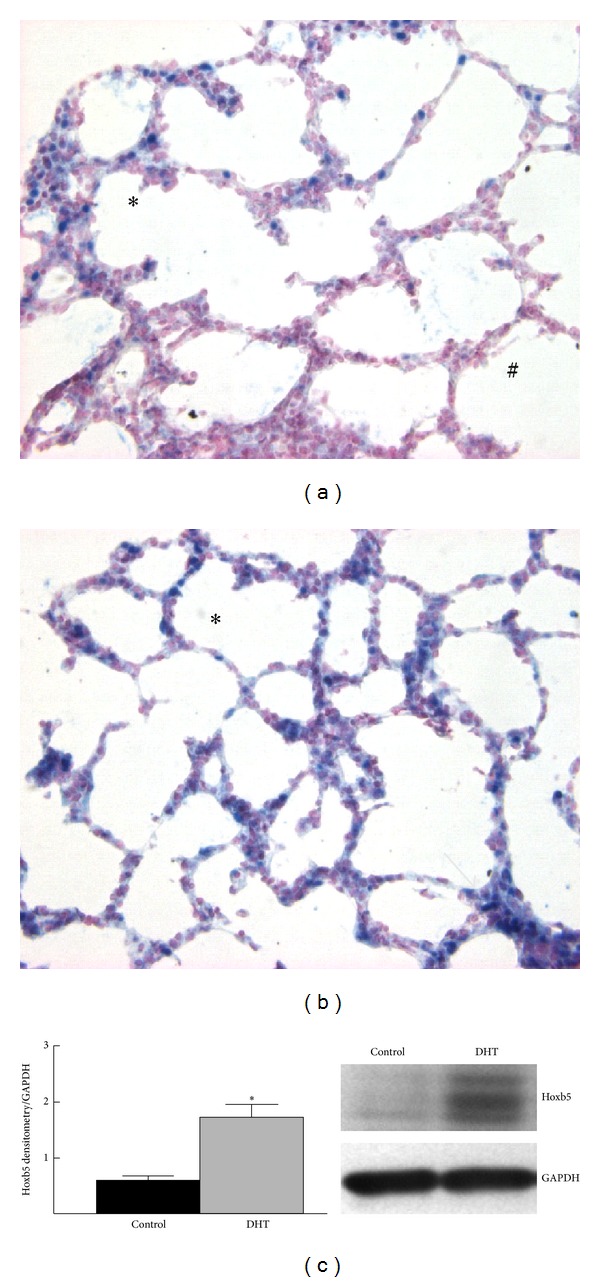
*In vivo* DHT treatment altered Hoxb5 cellular expression and protein levels. (a) Control E18 lungs exhibited a gradient of Hoxb5 localization (blue nuclear staining) with minimal mesenchymal expression in central lung (#) and increased expression in peripheral regions, especially around developing saccules (∗). (b) In DHT-treated lungs the Hoxb5 mesenchymal expression pattern lacked the normal central-to-peripheral gradient. Terminal sacs were less developed appearing rounder with few developing septa seen, indicating immaturity of airway patterning. 40x Mag in (a), (b). (c) Hoxb5 protein levels increased 3-fold with DHT treatment compared to control lungs. *P < 0.05, mean ± SEM, *N* = 4.

Similar to what we have previously reported, the effect of DHT on branching was not dependent on the fetal gender [18]. Therefore we did not analyze our *in vivo* results according to sex. In E18 control lungs the pattern of Hoxb5 expression was as we have previously reported, namely, a gradient of Hoxb5 mesenchymal localization that increased from central to peripheral lung (Figure 5(a)). Expression was greatest in mesenchymal cells immediately adjacent to developing airways at the periphery of the lung [8, 9]. Exposure to DHT from E11 to E18 (Figure 5(b)) altered this pattern. Hoxb5 expression was seen throughout lung mesenchyme with a loss of the central-to-peripheral gradient. Morphologically the developing saccules appeared more simplified with less well-developed septae. Consistent with this diffuse mesenchymal localization of Hoxb5, protein levels for Hoxb5 were significantly increased after *in vivo* DHT exposure (Figure 5(c)). 

**Figure 6:**
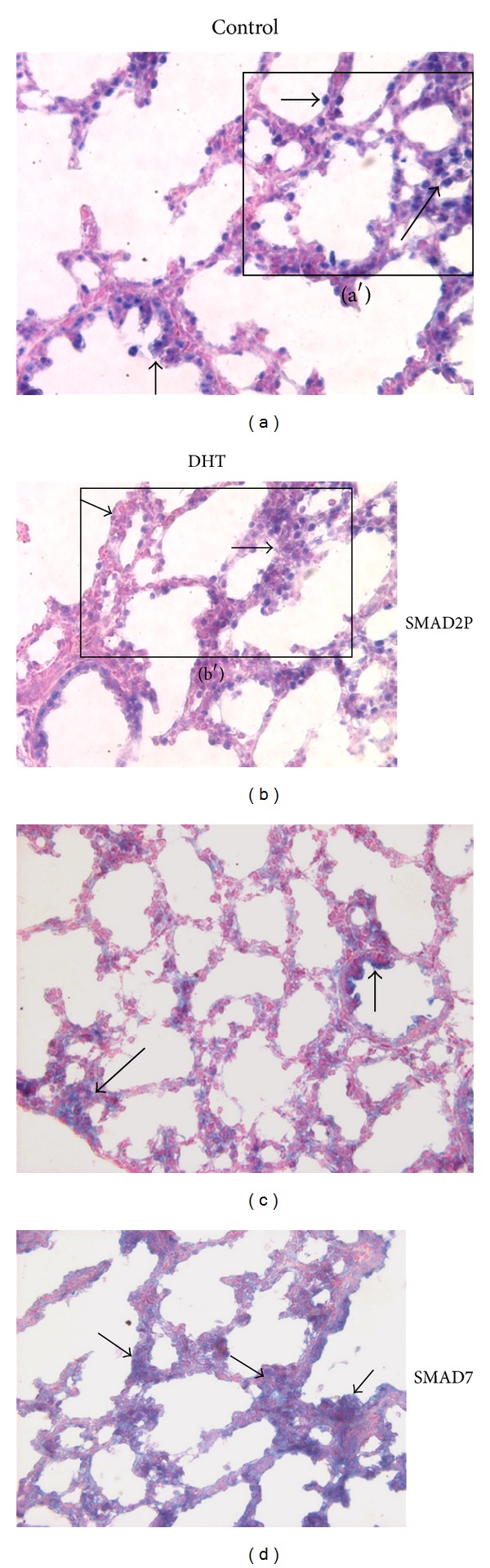
*In vivo* DHT treatment changed the spatial distribution of SMAD2P and SMAD7 proteins in E18 fetal mouse lungs. (a, a′, b, b′) SMAD2P; (c, d) SMAD7. Square inset in (a, b) represents areas of enlargement in (a′, b′). SMAD2P nuclear staining was seen in control (a, a′) and DHT (b, b′) treated E18 lungs in mesenchymal cells (long arrows) and epithelial cells (short arrows). However, epithelial (short arrows) and mesenchymal (long arrows) SMAD2P appeared less evident in DHT-treated lungs (b, b′). In control (c), SMAD7 was seen mostly in clusters of mesenchymal cells at the periphery of the lung (long arrow in (c)) and also in bronchiolar epithelial cells (short arrow in (c)). In DHT-treated lungs (d) the mesenchymal staining for SMAD7 (long arrows in (d)) was more diffuse, whereas bronchiolar epithelial cell (short arrows in (d)) staining was similar to controls. Figures are representative tissue sections from 3 lungs at each condition from 3 experiments; 40x Mag.

To determine if *in vivo* androgen exposure modulates TGF*β* signaling through changes in SMAD regulation, we evaluated the protein expression patterns of SMAD2P and SMAD7 in control and DHT-treated lungs. Nuclear expression of SMAD2P appeared less evident in mesenchyme and epithelial cells of DHT-exposed lungs (Figures 6(a), 6(a′), 6(b), and 6(b′)). SMAD7 was mainly expressed in mesenchyme and epithelial cells around and in peripheral airways in control lungs (Figure 6(c)). However, after DHT exposure, SMAD7 protein expression appeared more diffuse in mesenchyme throughout the entire lung (Figure 6(d)). 

## 4. Discussion

In this study we have shown for the first time a connection between androgens and the Hox protein Hoxb5, demonstrating that these factors interact to contribute to normal airway development and patterning, and this mechanism possibly occurs via regional changes in the cellular expression pattern of specific SMAD proteins. Hox genes closely related to Hoxb5 have important roles in other branching organs. For example, salivary gland branching in *Drosophila melanogaster* is mediated through levels of Scr (sex combs reduced), the drosophila homolog of Hoxb5 [31, 44]. In the human prostate gland Hoxb genes paralogous to Hoxb5 are required for control of androgen effects on normal gland development and function [24, 25]. The effects of androgens on type II cell maturation in developing lung are believed to be mediated by action on lung fibroblasts and subsequent changes in fibroblast-epithelial cell communication [19, 22, 45, 46]. This information along with our previous work showing a prominent effect of Hoxb5 mesenchyme expression on airway branching in mouse and human led us to consider the possibility that at least some of the effects of androgens in developing lung may be mediated by Hoxb5 in mesenchyme [9, 10]. This is the first report to our knowledge demonstrating that DHT mediates the localization and protein levels of a Hox protein in the developing lung.

We previously showed that Hoxb5 protein levels decrease earlier in lung development in female than in male fetuses [8]. These current findings add new physiologic insight into the potential consequences of this sexual dimorphism in fetal pulmonary Hoxb5 expression. The ability of DHT to alter airway branching was dependent on Hoxb5 protein expression. Therefore, our results indicate that the effect of androgens on developing lung in males is likely mediated at least in part through Hoxb5 protein expression. We cannot determine from this study if the effect of DHT on Hoxb5 expression is direct or indirect. Androgens act via receptor mediated mechanisms with activated androgen receptors altering gene transcription. However, androgens can also exert their actions by altering signal transduction within the cytoplasm [47]. The immediate Hoxb5 promoter region is not known to contain binding sites for androgen receptors. However, Hox gene promoters often extend into distant DNA sequences, including sequences within Hox genes 5′ to the Hox gene of interest [1]. Activated androgen receptors also dimerize with estrogen receptors to regulate gene transcription. Estrogen receptors regulate Hox gene expression. The Hoxb5 promoter has reported binding sites for estrogen receptor alpha. This information suggests a possible mechanism by which DHT treatment could transcriptionally upregulate Hoxb5 in our study [48–50].

Some of the effects of androgens in developing lung are also mediated through changes in TGF*β* signaling [19, 27], which can be modulated by Hox proteins [32, 42]. Therefore, we wanted to determine if the DHT effect on Hoxb5 expression altered the spatial localization of key TGF*β* effector and inhibitory SMAD proteins that help control lung morphogenesis, similar to the events seen during branching morphogenesis of the salivary gland [31]. Indeed, we found that *ex vivo* DHT treatment of mouse fetal lungs limited SMAD2P cellular localization whose function is important to activation of normal TGF*β* signaling. Changes in spatial localization of a protein is a significant determinant of changes in its functional role [3]. The ability of androgens to affect SMAD2P cellular expression pattern depended on a concomitant upregulation of Hoxb5 protein expression. This reveals that the effect of androgen on TGF*β* signaling is in part mediated by Hoxb5 mesenchymal expression. Our *in vivo* results are also consistent with this mechanism. This change in SMAD2P likely involves intermediate proteins or cellular events controlling phosphorylation of SMAD2, as we did not see changes in total SMAD2 (data not shown) with Hoxb5 manipulation or DHT treatment [51]. The changes we observed in SMAD2P and SMAD7 cellular localization with downregulation of Hoxb5 suggest that Hoxb5 in lung mesenchyme mediates the balanced effects of inhibitory and effector SMADs (SMAD7 and SMAD2P, resp.). This mechanism whereby Hoxb5 can locally modulate TGF*β* activity in lung appears to be similar to that observed between the *Drosophila Scr* (homolog of Hoxb5) and *dpp* (homolog of TGF*β*) genes during salivary gland development. In salivary gland, Scr regionally restricts cellular signaling by activated dpp. Conversely, dpp prevents widespread expression of Scr and excessive salivary gland development [31].

TGF*β* and Hox gene family members collaborate with each other to fine tune downstream gene expression [31, 52, 53]. Therefore, we sought to determine if the androgen-induced changes in TGF*β* signaling were upstream or downstream of Hoxb5. Our data suggest that TGF*β* signaling through SMAD2P may partially influence the Hoxb5 protein cellular expression pattern within lung mesenchyme. We also found that Hoxb5 inhibition altered mesenchymal cell localization of SMAD7. This change in SMAD7 likely contributed to the decrease in SMAD2P. Unexpectedly, chronic *in vivo* DHT exposure, while increasing Hoxb5 expression, also led to more diffuse SMAD7 mesenchymal staining. This chronic DHT exposure may have provided time for enhancement of a regulatory feedback pathway. The continued presence of inhibitory SMAD7 may therefore cooperate with Hoxb5 to limit SMAD2P effects. This is similar to the mechanism described for Hoxc8 and SMAD6, whereby SMAD6 increases the repressor function of Hoxc8 and in other circumstances Hoxc8 can repress SMAD6 function [30, 32].

In conclusion, our study has uncovered for the first time a novel mechanism by which androgen effects on lung branching morphogenesis are partially mediated by Hoxb5 protein regulation and that this level of regulation occurs partially through changes in cellular localization of SMAD2P and SMAD7. This ability of androgen signaling to maintain expression of Hoxb5 protein likely contributes to the known delayed airway epithelial maturation in male infants but positively contributes to the increased airway arborization and ultimate increased lung capacity in males compared to females.
